# Tidally driven remelting around 4.35 billion years ago indicates the Moon is old

**DOI:** 10.1038/s41586-024-08231-0

**Published:** 2024-12-18

**Authors:** Francis Nimmo, Thorsten Kleine, Alessandro Morbidelli

**Affiliations:** 1 Department of Earth and Planetary Sciences, University of California Santa Cruz, Santa Cruz, CA USA; 2 Max Planck Institute for Solar System Research, Göttingen, Germany; 3 Collège de France, CNRS, PSL University, Sorbonne University, Paris, France; 4 Laboratoire Lagrange, Université Cote d’Azur, CNRS, Observatoire de la Côte d’Azur, Boulevard de l’Observatoire, Nice, France

**Keywords:** Early solar system, Geochemistry, Early solar system

## Abstract

The last giant impact on Earth is thought to have formed the Moon^1^. The timing of this event can be determined by dating the different rocks assumed to have crystallized from the lunar magma ocean (LMO). This has led to a wide range of estimates for the age of the Moon between 4.35 and 4.51 billion years ago (Ga), depending on whether ages for lunar whole-rock samples^2–4^ or individual zircon grains^5–7^ are used. Here we argue that the frequent occurrence of approximately 4.35-Ga ages among lunar rocks and a spike in zircon ages at about the same time^8^ is indicative of a remelting event driven by the Moon’s orbital evolution rather than the original crystallization of the LMO. We show that during passage through the Laplace plane transition^9^, the Moon experienced sufficient tidal heating and melting to reset the formation ages of most lunar samples, while retaining an earlier frozen-in shape^10^ and rare, earlier-formed zircons. This paradigm reconciles existing discrepancies in estimates for the crystallization time of the LMO, and permits formation of the Moon within a few tens of million years of Solar System formation, consistent with dynamical models of terrestrial planet formation^11^. Remelting of the Moon also explains the lower number of lunar impact basins than expected^12,13^, and allows metal from planetesimals accreted to the Moon after its formation to be removed to the lunar core, explaining the apparent deficit of such materials in the Moon compared with Earth^14^.

## Abstract (web summary)

Lunar rock and zircon ages were reset by a remelting event driven by the Moon’s orbital evolution, reconciling existing discrepancies in estimates for the formation time of the Moon and the crystallization time of its magma ocean.

## Main

An age for the Moon can be determined by dating a process that results in chemical fractionation of parent and daughter elements of a suitable radionuclide chronometer and that can be closely linked to formation of the Moon itself. One such process is the crystallization of the lunar magma ocean (LMO), which led to density-driven separation of early mafic cumulates that sank to the bottom from buoyant plagioclase-rich cumulates that floated to the top of the LMO, forming the ferroan anorthosites (FANs) that dominate the lunar crust^15^. Crystallization of the LMO produced a residual liquid referred to as KREEP (for strong enrichments in potassium, rare earth elements and phosphorus), the formation of which is frequently used to mark the end of the LMO’s solidification^15^. Ages for the distinct early- and late-formed products of the LMO are remarkably consistent and all give an age of approximately 4.35 billion years ago (Ga), including (1) the most reliable crystallization ages of FANs, (2) the ^147^Sm–^143^Nd and ^176^Lu–^176^Hf model ages of KREEP, (3) a whole-rock ^146^Sm–^142^Nd isochron of FANs, mare basalts (which formed by remelting of the LMO’s mafic cumulates) and KREEP, and (4) crystallization ages of the magnesian suite (Mg suite; which represent melts intruded into the earlier-formed anorthositic crust) (see summary of ages in ref. ^16^). These ages have been interpreted to reflect rapid crystallization of the LMO and late formation of the Moon at approximately 4.35 Ga (refs. ^2,4,16^). However, thermal evolution models predict a more protracted LMO solidification, and interpreting the aforementioned ages within the framework of such models leads to an estimated earlier Moon formation at 4.425 ± 0.025 Ga (ref. ^17^). Either way, these young proposed ages are problematic for two reasons. First, they are late compared with the predictions of most dynamical models of planet formation^11,18^. Second, they are inconsistent with the occurrence of rare lunar zircons with older ages^5,7^ and hafnium isotopic compositions indicative of derivation from a KREEP source that may have formed as early as about 4.5 Ga (ref. ^6^), implying that the Moon would have formed even earlier. Early Moon formation ages have been proposed based on an approximately 4.51-Ga rubidium–strontium model for volatile loss from the Moon^19^ and an approximately 4.52 Ga hafnium–tungsten (Hf–W) model age for lunar core formation^20^, but the veracity of both ages is debated^21,22^. Nevertheless, if the Moon did form early, then the clustering of approximately 4.35-Ga lunar ages must record a major magmatic event unrelated to the LMO^16^; one such possible event is a large impact, for instance, the one that formed the South Pole–Aitken (SPA) Basin^8^.

**Fig. 1:**
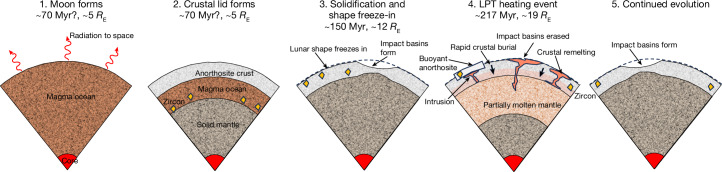
Hypothesized timeline of events. The timing of Moon formation and initial magma ocean freezing is uncertain. Zircons form during the final stages of LMO crystallization and are then transported upwards during eruptions. The Moon probably formed at around 5 *R*_E_ (ref. ^53^) and its shape froze-in at about 12 *R*_E_ (see text) and was not modified by the later tidal heating event (the LPT) at about 19 *R*_E_. However, the intense volcanism and reheating and/or burial associated with this event reset all crustal chronometers except for relict zircons, and erased pre-existing impact basins. Here time in million years (Myr) is counted forwards from the formation of the Solar System at 4,568 Myr before present.

Here we argue that the approximately 4.35-Ga age records an episode of tidal heating, and is not directly tied to either the formation of the Moon or the crystallization of the original post-impact magma ocean. Tidal heating has previously been proposed as an explanation for some of the Moon’s long-wavelength crustal features^23^. The tidally heated Moon was a ‘heat pipe’ body similar to Jupiter’s moon Io, in which heat is advected by hot melt intruding or erupting at the surface, rather than being conducted^24,25^. In this picture, partial melt percolates rapidly through the lunar mantle, causing widespread isotopic re-equilibration. The continued eruption of material prevents the development of a true magma ocean^26^ and results in rapid burial and/or reheating of the crust. As shown below, these processes should generally result in thermal resetting of the isotopic systems frequently used to date lunar samples, perhaps apart from those in some near-surface zircons. As such, a tidally driven remelting event at about 4.35 Ga resolves existing lunar chronological paradoxes and provides information on how tidal dissipation in the Earth has varied over time. Figure 1 summarizes our predicted timeline of events.

## Remelting during the Laplace plane transition at 4.35 Ga

Three possible episodes of tidal heating of the Moon can be identified: the evection resonance, at approximately 8 Earth radii (*R*_E_)^27–29^; the Laplace plane transition (LPT), at 16–22 *R*_E_ (ref. ^9^) and the associated inner and outer 3:2 resonances^30^; and the Cassini state transition, at 30–34 *R*_E_ (ref. ^31^). These resonances occur, respectively: when the Moon’s orbit precession period equals one year; when the effects of the Sun and Earth on the Moon’s orbital precession are equal; and when the lunar spin and orbit precession periods are equal. Of these transitions, the Cassini state transition occurs at the largest semi-major axis and thus the magnitude of tidal heating is low. Typical values are less than 0.1 W m^−2^ (ref. ^32^), which are unlikely to trigger widespread melting of the Moon. For reasons explained below, the Moon’s passage through the evection resonance is unlikely to be the main driver for remelting, simply because, to occur at 4.35 Ga, it would require the early Earth to be very non-dissipative, even less so than Jupiter, which is implausible. For these reasons, we focus here on the LPT, which was originally proposed to explain the high inclination of the Moon’s orbit^9^.

The peak tidal heating rate during the LPT is estimated to be 10^14^–10^15^ W (3–30 W m^−2^) for a few to several tens of million years^9,30,33^. The primary reason for the large energy release is that a high orbital eccentricity leads to strong tidal heating and a rapid decrease in the lunar semi-major axis. The heat flux range during the LPT may be compared with the present-day tidal heat production rate in Io of about 2.5 W m^−2^ (ref. ^34^), indicating that the Moon experienced Io-like or larger heat fluxes during the LPT.

**Fig. 2:**
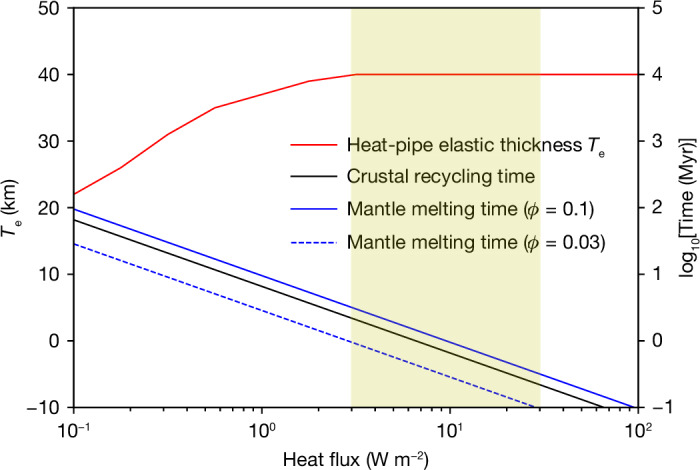
Recycling and melting timescales and elastic thickness as a function of heat flux. The shaded box denotes the inferred tidal heat flux during the LPT^9^. A heat-pipe Moon can retain a thick elastic layer while recycling the entire crust and remelting the entire mantle in a time short compared with the few to tens of million years duration of a tidal heating event. The crustal thickness is taken to be 40 km and *ϕ* is the mean mantle melt fraction. Further details can be found in Methods. Source Data

These high heat fluxes imply prodigious mantle melting and volcanism. Assuming that the tidally generated heat is removed from the mantle via advection of melt, we can calculate how long it takes for the entire mantle to be fluxed through the melting region (Methods). Figure 2 shows that for the estimated LPT heat fluxes, this timescale is of order a few million years, depending on the melt fraction. Thus, over the duration of the LPT heating event^9,30^, we expect the entire mantle to be partially remelted a few times. However, because of the rapid melt removal, we do not expect an actual magma ocean to form^26^.

## Crustal recycling and zircon resetting

Melt produced during the LPT may be either primarily erupted at the surface or intruded within the crust; different regions of the Moon will be dominated by intrusion or extrusion depending on the local density contrast between melt and crust^35^. Pre-existing anorthositic crustal blocks, being low density, are likely to be intrusion dominated.

For end-member cases where all melts are erupted to the surface, crustal material is continually buried and advected downwards by erupting lavas. Sufficiently deep burial will result in thermal resetting and, eventually, remelting. The characteristic timescale to reset the entire crust *t*_o_ is simply *t*_o_ = *h*_c_/*u*, where *h*_c_ is the crustal thickness and *u* is the areally averaged vertical melt velocity. Figure 2 plots the crustal recycling time as a function of the heat flux and shows that for the LPT range of 3–30 W m^−2^, this time is about 0.1–1 Myr (Methods). Given the likely LPT duration of a few to tens of million years^9,30,33^, complete recycling is expected, so that the final crustal ages recorded in these regions will simply be the time at which the LPT-driven recycling ceased.

**Fig. 3:**
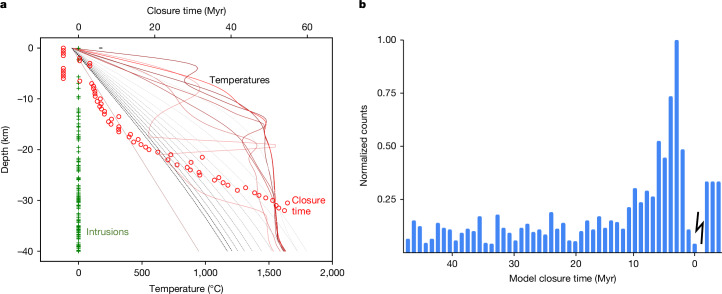
Effect of intrusions on zircon Pb–Pb closure ages. The tidal heating and intrusions begin at 0 Myr and end at 3.5 Myr. **a**, A single realization of our thermal model. The solid red lines show temperatures at equally spaced times from 0 Myr to 3.5 Myr; the grey and black dashed lines from 5 Myr to 55 Myr. The green crosses show the depths of individual intrusions, where here the intrusion scale height is 20 km. The red circles denote the Pb–Pb closure time for 50-μm-radius zircons. Negative values indicate zircons that are never reset. Here we assume a melt advection velocity *u* = 6 cm yr^−1^ and the intrusion thickness is 2 km (Methods). **b**, Histogram of Pb–Pb closure time from 30 realizations, where the count is normalized to the largest value. Negative closure times mean that the zircons were never reset, so the distribution of ages is arbitrary. It is noted that the pronounced peak is approximately coincident with the end of the tidal heating event. Source Data

For areas that experience intrusive rather than extrusive volcanism, we created a simple conductive thermal evolution model to study the effects of multiple intrusions, and track how these intrusions reset the ages recorded by rocks and zircons (Methods). Figure 3a shows snapshots from an example model. The crust heats up while intrusions (green crosses) are added over a period of 3.5 Myr appropriate for the LPT (red lines), and then subsequently cools (grey and black lines). Zircons (red circles) record a lead–lead (Pb–Pb) closure time (relative to the model start time) depending on their cooling history. Some near-surface zircons (negative ages) are never reset because they cool too fast; deep-seated zircons cool slowly and thus record a wide range of closure times. In between, there is a pile-up of ages around 3–5 Myr (near the time heating ends in this particular model), because this region cools rapidly once the heating episode ends. Figure 3b plots a histogram of model zircon Pb–Pb closure times using 30 realizations, similar to Fig. 3a. We find that there is a peak in the distribution at 3–5 Myr, and a small fraction (about 12%) of zircons are not reset at all. These model results—a narrow peak^8^ and a few ancient zircons^5,7^—strongly resemble the characteristics of actual lunar zircons.

Apart from the zircons, tidal heating during the LPT resolves other paradoxes in the chronology of the Moon. First, mare basalts, FANs and KREEP-rich samples plot along a single ^146^Sm–^142^Nd isochron^4,36^, which has been interpreted to indicate that these samples were in isotopic equilibrium at approximately 4.35 Ga. These samples originate from various depths within the Moon, ranging from the first tens of kilometres down to a few hundreds to 1,000 km (refs. ^37,38^). Consequently, attaining isotopic equilibrium for such a significant volume of the Moon is only possible by large-scale advection of melt. This, combined with the widespread occurrence of approximately 4.35-Ga ages among lunar samples, has until now been ascribed to a late formation of the Moon and rapid solidification of the LMO^4,16^. However, thermal models suggest a longer-lived LMO, which should have produced crystallization products with distinct ages^17^. A tidal heating event explains both the preponderance of approximately 4.35-Ga lunar ages^16^ and isotopic equilibrium across large portions of the Moon^4^, given that this event was short-lived (a few tens of million years at most^9,30,33^) with respect to the uncertainties of the lunar ages.

**Extended Data Fig. 1:**
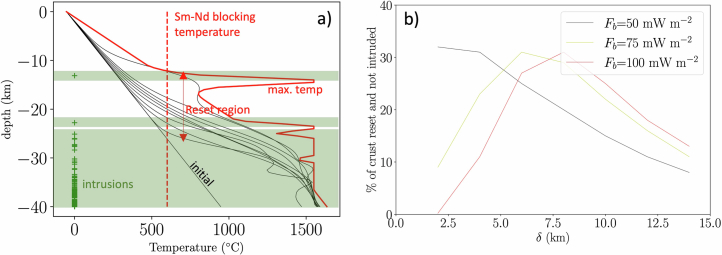
Result of intrusion model on Sm-Nd ages. a) A realization of the same thermal model as in Fig. 3 but with δ = 4 km (intrusions concentrated towards the base of the crust). The red line shows the maximum temperature experienced at each depth. The green boxes show where intrusions are added. The mid-crust has regions that are reset (maximum temperature exceeds Sm-Nd blocking temperature) but are not actually intruded. The blocking temperature of the Sm-Nd system in plagioclase is from ref. ^70^. b) Fraction of crust reset but not intruded as a function of scale height δ and basal heat flux *F*_*b*_. Source Data

Second, the lunar Mg suite appears to derive from distinct reservoirs of the LMO, including mafic cumulates from the early stages of LMO solidification, plagioclase-rich cumulates similar to FANs, and KREEP. Thus, the Mg suite must have formed by remelting after initial LMO crystallization^37^. Crystallization ages of Mg-suite rocks also cluster around approximately 4.35 Ga, which so far has been explained by remelting due to cumulate overturn immediately after rapid LMO solidification^16^. However, tidal remelting of the Moon will result in intrusion of melt into any pre-existing crust, naturally explaining the close temporal link between the Mg-suite rocks and the earlier-formed FANs. Our models show that these intrusions result in resetting of ages for nearby FANs (Extended Data Fig. 1), consistent with the indistinguishable ages of FANs and Mg-suite rocks. Moreover, this scenario permits the preservation of older ages for those FANs that remained more distant from any intrusion, although the current evidence for such rocks is weak^39^.

Although in principle the SPA Basin^8^ or the older, predicted Procellarum Basin^40^ could have caused the resetting event recorded in the approximately 4.35-Ga lunar ages, recent models and analysis documented in Methods do not provide strong support for these ideas. In our model, the SPA Basin should be younger than 4.35 Ga, because otherwise it would have been erased.

## Implications for the early evolution of the Moon

A tidally induced remelting of the Moon at approximately 4.35 Ga is consistent with several prominent features of the Moon, including the survival of the Moon’s fossil bulge, the absence of ancient impact basins, and the disparate late accretionary histories of Earth and the Moon. The Moon appears to have ‘frozen in’ its shape at some earlier epoch when it was closer to the Earth and had different orbital or rotational characteristics^10,41^. Although the details are controversial, freezing in this fossil bulge requires the development of a rigid elastic layer, which must not be disrupted by a later tidal heating event. Importantly, one of the characteristics of an extrusive heat-pipe body is that the bulk of crust at any time is cool and rigid^24^, thus allowing a fossil bulge to persist.

A previous study^10^ found that the fossil bulge can be explained if a 12.8-km-thick elastic layer developed when the Moon was at a distance of 13 *R*_E_, whereas an elastic thickness *T*_e_ of 25 km requires a semi-major axis of 16 *R*_E_. Thus, the fossil figure was probably established before the LPT. If *T*_e_ had decreased below these values subsequently, the fossil bulge would have been reduced. However, Fig. 2 shows that the *T*_e_ inferred for an extrusive heat-pipe Moon during the LPT (solid red line) can be up to 40 km and thus permit fossil-bulge survival. Intrusions would reduce this value but still permit a cold, rigid layer to persist^25^.

Although the Moon is canonically cratered, with about 50 impact basins, dynamical models suggest that it should host more^12^. One recent study suggested that all basins and craters forming before 4.35–4.41 Ga were erased^13^. Some ancient impacts may have gone unrecorded because they occurred while a subsurface magma ocean was present^42^. But remelting in the mantle and the massive volcanism and resurfacing associated with the approximately 4.35-Ga tidal heating event provides an alternative way of erasing the Moon’s earlier bombardment history and explaining the observed basin and crater population.

Finally, a puzzle concerning the Moon is the much lower concentration of highly siderophile elements (HSEs) in its mantle compared with Earth^14^. Previous explanations for this feature include disproportional late accretion of large objects to Earth^43^ and late HSE removal from the lunar mantle during slow magma ocean crystallization terminated by mantle overturn^12^. Our remelting model offers an alternative, as follows. After the original magma ocean crystallized, subsequent impacts will have stranded metal in the lunar mantle^44^. A later remelting of the mantle will have remobilized this metal, which would scavenge HSEs as they descend to the lunar core. If the lunar mantle lost all the HSEs accumulated before 4.35 Ga, the model lunar HSE concentrations delivered by subsequent impacts match those measured, assuming 30% retention of impact material on the Moon^45^. It is striking that this argument, based solely on dynamical simulations, yields an onset time for HSE retention of approximately 4.35 Ga (refs. ^12,13,45^), consistent with our remelting hypothesis.

## Implications for the age of the Moon

Interpretation of the approximately 4.35-Ga lunar ages as a result of tidal heating rather than original LMO crystallization implies that the Moon formed earlier. As the LPT occurs at a particular semi-major axis (16–22 *R*_E_)^9^, by tying it to a remelting event at 4.35 Ga we can make inferences about the Moon’s early orbital evolution. The primary driver of lunar migration is tidal dissipation in Earth, parameterized by the dissipation factor *Q*_E_. Early migration was rapid and probably on a timescale comparable to that on which Earth was evolving during its recovery from the Moon-forming impact. Consequently, predicting *Q*_E_ from first principles is challenging and depends on poorly known factors such as the timescale for Earth’s magma ocean crystallization and the thickness and duration of any early atmosphere^46,47^.

**Fig. 4:**
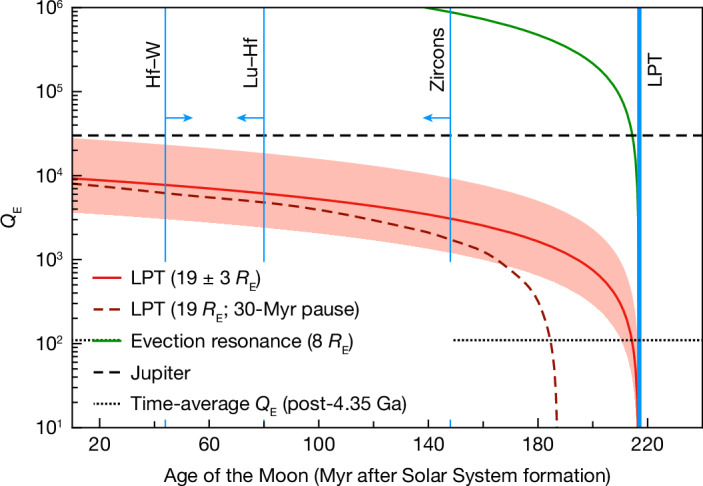
Constraints on early Earth dissipation. Dissipation factor *Q*_E_ shown as a function of Moon formation time for the Earth to reach 19 ± 3 *R*_E_ at 4.35 Ga (red line). The dashed red line includes a pause at the LPT of 30 Myr (see text). The horizontal black lines indicate two constraints on *Q*_E_: the mean value after 4.35 Ga (see text); and the measured value of *Q* for Jupiter^49^, which we take to be an upper bound for Earth. The vertical blue lines denote the time of the LPT, Pb–Pb ages of rare individual zircons^5,7^, a Lu–Hf model age for KREEP formation derived from lunar zircons^6^, and the Hf–W model age for core formation on Earth, which provides the earliest possible time of Moon formation^52^. Source Data

Nonetheless, we can deduce an average *Q*_E_ applicable to this period of lunar evolution (Methods). The red line in Fig. 4 shows the trade-off between the time the Moon formed and the mean *Q*_E_ required for it to reach 19 *R*_E_ at 4.35 Ga. The uncertainty in the LPT distance (±3 *R*_E_) is indicated by the red shading. An early formation time allows a less dissipative Earth (higher *Q*_E_). The Moon may have stalled at the LPT for some tens of million years^9,30,34^, but Fig. 4 shows that this has very little effect on *Q*_E_ except if the Moon formed late (which is ruled out by the zircon ages; see below). The *Q*_E_ values derived are generally much higher (less dissipative) than the present-day solid Earth, for which *Q*_E_ ≈ 300 (ref. ^48^). However, they are lower than the *Q* of Jupiter, which has been measured to be roughly 3 × 10^4^ using astrometry^49^. One would not expect a solid silicate body, even if fully molten, to be less dissipative than a gas giant like Jupiter^50^, and so the *Q* of Jupiter can be taken as strict upper limit for *Q*_E_.

Figure 4 shows that the time interval between the formation of the Moon and the onset of the LPT is uncertain; it could be as much as about 200 Myr or as short as about 10 Myr. The latter interval is consistent with long-term orbital evolution models^51^, where the Moon reaches 20 *R*_E_ about 10 Myr after formation. However, also shown in Fig. 4 are the ages of the oldest lunar zircons as well as the Lu–Hf model age of KREEP formation as determined on lunar zircons. As these zircons formed after substantial differentiation of the Moon^6^, the Moon must have formed before these zircons crystallized at approximately 4.43 Ga, that is, at least approximately 80 Myr before the LPT. Conversely, a lower limit for the time of Moon formation is provided by the two-stage Hf–W model of core formation in Earth of 4.533 Ga, which provides the earliest time at which core formation can have ceased^52^. As the last core formation event on Earth is thought to have been triggered by the Moon-forming impact, this model age also provides the earliest time at which the Moon can have formed, which is about 180 Myr before the LPT.

Although the poor knowledge of *Q*_E_ limits our ability to precisely date the formation of the Moon using the time of the LPT, our model strongly suggests that the Moon formed much earlier than 4.35 Ga, probably in the range of 4.43–4.53 Ga. Dynamical models show that terrestrial planet formation is sufficiently stochastic to allow for a Moon-forming event as late as about 200 Myr, even starting from a concentrated distribution of material around 1 AU (ref. ^11^). However, such a protracted phase of terrestrial planet formation typically leads to systems that are dynamically overexcited, and the amount of material accreted by Earth after formation of the Moon is too small to account for the abundances of HSEs in Earth’s mantle^11,18^. Formation of the Moon at approximately 4.5 Ga would solve these problems, but in this case the Moon would have accumulated many more impact basins and more late-accreted material than observed^12,13^. Thus, our proposal of an early Moon formation followed by a late, tidally driven remelting appears a likely way of reconciling these apparently contradictory observations.

## Methods

### Heat-pipe Moon

The advection–diffusion equation in a two-dimensional Cartesian geometry with no internal heating is written^24^

$$\frac{\partial T}{\partial t}=\kappa \frac{{\partial }^{2}T}{\partial {z}^{2}}-u\frac{\partial T}{\partial z}$$

where *T* is temperature, *κ* is the thermal diffusivity, *u* is the downwards velocity of the crust due to burial and *z* is positive downwards. In steady state, the solution is

1 $$T={T}_{{\rm{s}}}+\frac{({T}_{{\rm{b}}}-{T}_{{\rm{s}}})}{({{\rm{e}}}^{\frac{u}{\kappa }{h}_{c}}-1)}\left({{\rm{e}}}^{\frac{u}{\kappa }z}-1\right)$$

where *T*_s_ is the surface temperature and *T*_b_ is the temperature at the base of the crust (thickness *h*_c_). It is noted that this expression reduces to the standard conduction equation in the limit that *u* is small. The conductive heat flux at the base of the crust is *F* = (*T*_b_ − *T*_s_)*u*/*κ*, which can be rewritten as *ρC*_*p*_*u*(*T*_b_ − *T*_s_), the advective heat flux (the two must balance in steady state). Here *ρ* is the density and *C*_*p*_ is the specific heat capacity, modified to include a latent heat effect (that is, we augment the usual specific heat capacity with a term *L*/(*T*_b_ − *T*_s_) where *L* is the latent heat).

The isotherm defining the base of the elastic layer in oceanic mantle material on Earth is at about 720 K (ref. ^54^). We use this isotherm and equation (1) to determine the elastic thickness as a function of the heat flux. For a specified heat flux *F*, we can also solve for *u* and thus derive the overturn time *t*_c_/*u*. Thus, if *F* = 10 W m^−2^, we obtain *u* = 0.06 m yr^−1^ and a crustal overturn time of 0.67 Myr.

The mantle melting timescale may be derived as follows. The surface flow rate of material is 4π${R}_{M}^{2}$*u*, and if the melt fraction is *ϕ* then the rate at which mantle material is fluxed through the melting zone is 4π${R}_{M}^{2}$*u*/*ϕ*, where *R*_M_ is the lunar radius (1,740 km). The total volume of the mantle is approximately that of the whole Moon, 4/3π${R}_{M}^{3}$. Thus, the timescale to flux the whole mantle through the melting zone is *R*_M_*ϕ*/3*u*. If *ϕ* = 0.1 and *u* = 0.1 m yr^−1^, then the timescale is 0.58 Myr, comparable to the crustal overturn timescale.

The parameters adopted are as follows: *ρ* = 2,600 kg m^−3^, *k* = 2 W m^−1^ K^−1^ (ref. ^17^), *L* = 450 kJ kg^−1^, *C*_*p*_ = 1,200 J kg^−1^ K^−1^, *T*_s_ = 220 K and *T*_b_ = 1,500 K. In our baseline models, we take *h*_c_ = 40 km (ref. ^55^). It has been suggested previously that the early Moon was a heat-pipe body^56^, but not in the context of a tidal heating event.

### Intrusive resetting

We solve a simple static one-dimensional finite-difference heat-conduction equation in which intrusions are added at intervals. To take into account the random nature of intrusive behaviour, we assign a probability distribution for the height above the base of the crust *z*′ at which the intrusion occurs, where the probability is proportional to exp(−*z*′/*δ*), with *δ* an user-specified scale height. A low value of *δ* means that intrusions are concentrated towards the base of the crust. For simplicity, we assume a single characteristic intrusion thickness Δ*d* (an integer multiple of the grid spacing Δ*z*) and a characteristic time interval between intrusions Δ*t*. Once Δ*d* is specified, Δ*t* is then determined by the requirement that Δ*d*/Δ*t* = *u*, where *u* is calculated as described above.

At intervals Δ*t*, we intrude an intrusion of thickness Δ*d* at a randomly chosen grid point in the crust. The intruded material is given an initial temperature of *T*_m_ and the surface temperature *T*_s_ is kept constant. The basal heat flux *F*_b_ is also kept constant at a value appropriate for the Moon before the heating event; the assumption is that tidal heat produced in the mantle is being transported by advection (melt ascent) rather than conduction. As intrusions proceed, the basal temperature will increase. Tidal heating starts at model time zero, ends after 3.5 Myr and we then continue the model up to 55 Myr. The finite-difference timestep is set to be 0.3Δ*z*^2^/*κ* to satisfy the Courant criterion, with Δ*z* = 0.5 km. The thermal parameters are the same as for the heat-pipe model (above); other nominal parameter values are: *T*_m_ = 1,550 °C, *F*_b_ = 50 mW m^−2^ and Δ*d* = 2 km.

We use the same model to investigate the extent to which heating by the intrusions results in resetting of Pb–Pb zircon ages. Here we implement a simple model for diffusion to track the time at which diffusive loss effectively ceases; this time will give the age recorded.

For a zircon with constant diffusivity *D*, the time *τ* it takes for diffusive loss to penetrate a distance *p* into the crystal is given by *p* = (π^2^*Dτ*)^1/2^, where π^2^ is a factor appropriate for our (assumed spherical) crystal^57^. In reality, *D* is temperature dependent. We therefore differentiate the constant-*D* relationship to obtain

$$\Delta p\approx \frac{{\rm{\pi }}}{2}{\left(\frac{D}{t}\right)}^{1/2}\Delta t$$

Here Δ*p* is the change in penetration distance over a time interval Δ*t*. As long as *D* is changing slowly, we can use this expression to determine how *p*(*t*) increases with time. Once *p*(*t*) equals half the radius of the crystal, 7/8 of the crystal volume will have experienced Pb loss, resetting is assumed to have occurred and in the next timestep we restart the calculation setting *p* = 0 and *t* = Δ*t* (to avoid a singularity at the origin). In this manner, we can track when Pb loss effectively ceases. We assume that the diffusivity is given by *D*_0_exp(−*Q*/*RT*), where for Pb *D*_0_ = 0.11 m^2^ s^−1^ and *Q* = 550 kJ mol^−1^ (ref. ^58^) and *R* is the gas constant. Application of this approach with a cooling rate of 10 °C Myr^−1^ yields closure temperatures of 968 °C and 877 °C with zircon radii of 100 μm and 10 μm, respectively. These values compare favourably to the values of about 990 °C and 895 °C shown in Fig. 13 of ref. ^58^. In our nominal model, we assume a zircon radius of 50 μm.

**Extended Data Fig. 2:**
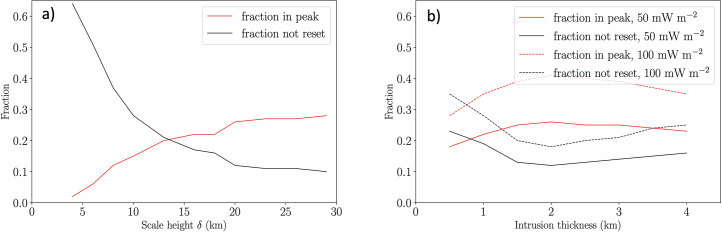
Sensitivity of zircon results to parameter variations. a) Fraction of zircons with Pb-Pb ages 3-6 Myr (i.e. as the heating episode ends) (red line) and fraction with Pb-Pb ages that are not reset (black line) as a function of the intrusion scale height δ, based on 30 realizations with F_b_ = 50 mWm^−2^ and *Δd* = 2 km. The peak in ages between 3-6 Myr becomes more pronounced as intrusions become more widely distributed throughout the crust (larger δ). b) As for a) but exploring the zircon fraction as a function of basal heat flux *F*_*b*_ and intrusion thickness *Δd*. Here δ = 20 km. Calculation techniques are described in Methods. Source Data

**Extended Data Fig. 3:**
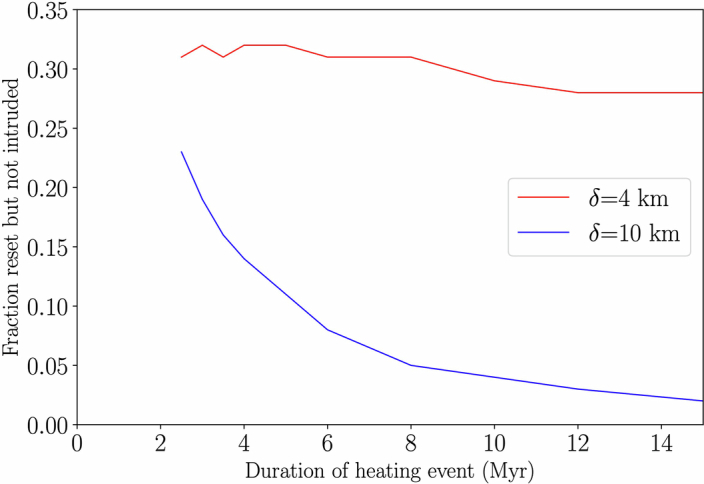
Sensitivity of thermally reset crust to duration of heating event. Sensitivity of fraction of crust reset (in terms of Sm-Nd ages) and not intruded, as a function of the duration of the heating event. The parameter δ is the scale height of the intrusions (small δ means intrusions are more clustered towards the base of the crust). Source Data

For a given depth within the Moon, we know how the temperature is evolving with time and can therefore calculate *D*(*t*) and the time at which the last resetting takes place for any zircons present at that depth. This approach is the basis of the zircon ages shown in Fig. 3, where a uniform initial distribution of zircons with depth is assumed. The results change minimally (<1%) if we double or halve the zircon radius. If the intrusions are more concentrated towards the base of the crust, the fraction of zircons not undergoing resetting increases, as expected (Extended Data Fig. 2). The same analysis for Hf (which diffuses more slowly) shows that around 30% of zircons are not reset for our nominal model parameters. Longer-duration heating events result in more rock resetting (Extended Data Fig. 3).

### Lunar orbital evolution

Dissipation in the Earth drives outwards evolution of the Moon, whereas dissipation in the Moon circularizes the Moon’s orbit and can also drive inwards orbital evolution^59^. Below we assume that dissipation in the Earth dominates. The semi-major axis evolution of the Moon *a* is then given by^60^:

2 $$\frac{{\rm{d}}a}{{\rm{d}}t}=3\frac{{k}_{2{\rm{E}}}}{{Q}_{{\rm{E}}}}\frac{m}{M}{\left(\frac{{R}_{{\rm{E}}}}{a}\right)}^{5}na$$

Here *k*_2E_ and *Q*_E_ are the tidal Love number and dissipation factor of the Earth, *m* and *M* are the mass of the Moon and Earth, respectively, *R*_E_ is the radius of the Earth and *n* is the mean motion of the lunar orbit. We take *M* = 6 × 10^24^ kg, *m* = 7.4 × 10^22^ kg, *R*_E_ = 6,400 km and *k*_2E_ = 0.97. The latter value is that appropriate for a strengthless (for example, molten) Earth, rather than taking the present-day value of 0.299, which is due to the rigidity of the present-day mantle^59^. A limitation of all existing models of the LPT^9,30,34^ is that they assume constant *Q* and *k*_2_ values; we anticipate that incorporating thermal–orbital feedbacks will shorten the period of tidal heating.

### The role of ancient impacts

We consider whether it is possible that a large impact that formed the SPA Basin or the older, predicted Procellarum Basin^40^ caused the resetting event recorded in the approximately 4.35-Ga lunar ages. Regarding the SPA Basin, there are three possibilities. The first is that ejecta from the SPA impact itself polluted the Apollo region, but models show that this does not take place^61^. Second, SPA melt-sheet material may have been redistributed by subsequent impacts^8^, but we find that the fraction of such material at the Apollo sites was only around 2% (see below). Third, the SPA Basin might have triggered mantle convection and melting^62^, but a potential problem with this model is that the volcanism it produces is long-lived and would not obviously generate the spike in ages that a short period of tidal heating does, and that is observed among the lunar ages. This model also implies melting focused on one hemisphere, whereas ours argues for global melting. Of note, the lunar meteorite Kalahari 009 shows a Pb–Pb age of 4.369 ± 0.007 Ga and based on chemical grounds is thought to derive from the lunar farside, consistent with our model of a global remelting event at around 4.35 Ga (ref. ^63^). As this event will probably have erased any pre-existing basins, we predict that the SPA Basin itself is younger than 4.35 Ga.

Finally, models of the impact that formed the putative Procellarum Basin^45^ show that the resulting impact melt is localized and would not be sufficient to cause the kind of global mixing and resetting that the lunar samples appear to require. Thus, current models do not support the idea of impacts being responsible for the resetting event.

### Redistribution of material from the SPA Basin

The Apollo sites will have received material originating from the SPA melt sheet and redistributed by subsequent impacts^8^. They will also have received material ejected from other regions of the Moon. We wish to compare the relative masses of these two contributions. The key factor is that the fraction of ejecta travelling with a particular velocity decreases as that velocity increases^64^; thus more distant impacts supply a lower fraction of ejecta material compared with nearer impacts.

Using the simple Maxwell model^65,66^, we can show that the volume of material *V*_s_ ejected at a radial velocity exceeding a specified value *u*_s_ ratioed to the total volume of material *V* ejected is given by

3 $$\frac{{V}_{{\rm{s}}}}{V}\approx {\left(\frac{g{R}_{{\rm{t}}}}{4Z(Z-2){u}_{{\rm{s}}}^{2}}\right)}^{3/2Z}$$

where *g* is the surface gravity, *R*_t_ is the transient crater diameter and *Z* is a constant. A larger transient crater produces a greater fraction of high-velocity material, but the sensitivity is weak^64^. The minimum radial velocity *u*_s_ required for a particle to travel a distance *s* is given by (*gs*/2)^1/2^.

We need to deduce the transient crater radius *R*_t_ from the observed crater radius *R*_f_. To do so, we use the scaling used in ref. ^67^ where

4 $${R}_{{\rm{t}}}={({R}_{{\rm{c}}}^{\xi }{R}_{{\rm{f}}})}^{1/(1+\xi )}$$

Here *R*_c_ is the simple-complex crater transition radius (9 km for the Moon) and *ξ* is a constant.

We use a catalogue of all impact craters exceeding 1–2 km diameter on the Moon^68^, a total of 1,296,795 excluding the SPA Basin itself. To calculate the volume of material ejected from a particular region by subsequent impacts, we use the following algorithm for each impact crater. (1) Determine whether the centre of the impact crater falls within the specified region (for example, the SPA melt sheet). (2) If it does, determine the great-circle distance *s* from the centre of the impact crater to the target location. (3) Calculate the minimum horizontal speed *u*_s_ required to achieve this distance. (4) Calculate the transient crater radius *R*_t_ associated with the measured final radius of the crater *R*_f_ using equation (4). (5) Use *u*_s_, *R*_t_ and equation (3) to calculate the volume of material ejected at a speed exceeding *u*_s_ compared with the total volume of material ejected.

This algorithm can be repeated for each crater observed to determine the total mass ejected from the specified region capable of reaching the target site. We perform this algorithm twice, once for craters within the SPA melt sheet and once for craters elsewhere. The ratio of the two answers gives a measure of what fraction of all material accumulating at the target site is derived from the SPA melt sheet. For our nominal parameter values, we find a value of 1.7%.

We follow ref. ^66^ and take *Z* = 2.71. Equation (3) then yields an exponent of 0.55, slightly lower than the range of 0.6–0.85 advocated by ref. ^64^. A higher value yields a lower value of *Z* and results in a smaller contribution from the SPA basin. For instance, if we take *Z* = 2.2, then the volume fraction is reduced to 1.4% compared with 1.7% for the nominal model. We use *ξ* = 0.22 to reproduce the relationship between the transient and final crater diameter derived by ref. ^69^. Using a lower value of *ξ* = 0.13 causes a slight reduction in the volume fraction deriving from the SPA Basin (1.3% compared with 1.7%). We take *g* = 1.6 m s^−2^ and use the location (0°, 0°) as an appropriate average of the Apollo site locations. Variations in longitude or latitude by ±10° change the volume fraction answer by less than 0.1%.

## Online content

Any methods, additional references, Nature Portfolio reporting summaries, source data, extended data, supplementary information, acknowledgements, peer review information; details of author contributions and competing interests; and statements of data and code availability are available at 10.1038/s41586-024-08231-0.

## Source data

Source Data Fig. 2

Source Data Fig. 3

Source Data Fig. 4

Source Data Extended Data Fig. 1

Source Data Extended Data Fig. 2

Source Data Extended Data Fig. 3

## Data Availability

The output used to produce the figures is available at 10.5061/dryad.kprr4xhdz. Source data are provided with this paper.

## Code availability

The codes used to generate results are available at 10.5281/zenodo.13968139.

## References

[1] Canup, R. M. & Asphaug, E. Origin of the Moon in a giant impact near the end of the Earth’s formation. *Nature* **412**, 708–712 (2001). 11507633 10.1038/35089010

[2] Borg, L. E., Connelly, J. N., Boyet, M. & Carlson, R. W. Chronological evidence that the Moon is either young or did not have a global magma ocean. *Nature* **477**, 70–72 (2011). 21849974 10.1038/nature10328

[3] Gaffney, A. M. & Borg, L. E. A young solidification age for the lunar magma ocean. *Geochim. Cosmochim. Acta* **140**, 227–240 (2014).

[4] Borg, L. E. et al. Isotopic evidence for a young lunar magma ocean. *Earth Planet. Sci. Lett.* **523**, 115706 (2019).

[5] Nemchin, A. et al. Timing of crystallization of the lunar magma ocean constrained by the oldest zircon. *Nat. Geosci.* **2**, 133–136 (2009).

[6] Barboni, M. et al. Early formation of the Moon 4.51 billion years ago. *Sci. Adv.* **3**, e1602365 (2017). 28097222 10.1126/sciadv.1602365 PMC5226643

[7] Greer, J. et al. 4.46 Ga zircons anchor chronology of lunar magma ocean. *Geochem. Persp. Let.* **27**, 49–53 (2023).

[8] Barboni, M. et al. High-precision U–Pb zircon dating identifies a major magmatic event on the Moon at 4.338 Ga. *Sci. Adv.* **10**, eadn9871 (2024). 39047092 10.1126/sciadv.adn9871 PMC11268413

[9] Cuk, M., Hamilton, D. P., Lock, S. J. & Stewart, S. T. Tidal evolution of the Moon from a high-obliquity, high-angular-momentum Earth. *Nature* **539**, 402–406 (2016). 27799656 10.1038/nature19846

[10] Matsuyama, I., Trinh, A. & Keane, J. T. The lunar fossil figure in a Cassini state. *Planet. Sci. J.* **2**, 232 (2021).

[11] Woo, J. M. Y., Nesvorný, D., Scora, J. & Morbidelli, A. Terrestrial planet formation from a ring: long-term simulations accounting for the giant planet instability. *Icarus* **417**, 116109 (2024).

[12] Morbidelli, A. et al. The timeline of the lunar bombardment: revisited. *Icarus* **305**, 262–276 (2018).

[13] Nesvorný, D. et al. Early bombardment of the moon: connecting the lunar crater record to the terrestrial planet formation. *Icarus* **399**, 115545 (2023).

[14] Day, J. M. D. & Walker, R. J. Highly siderophile element depletion in the Moon. *Earth Planet. Sci. Lett.* **423**, 114–124 (2015). 34465923 10.1016/j.epsl.2015.05.001 PMC8404368

[15] Warren, P. H. The magma ocean concept and lunar evolution. *Annu. Rev. Earth Planet. Sci. Lett.* **13**, 201–240 (1985).

[16] Borg, L. E. & Carlson, R. W. The evolving chronology of Moon formation. *Annu. Rev. Earth Planet. Sci.* **51**, 25–52 (2023).

[17] Maurice, M., Tosi, N., Schwinger, S., Breuer, D. & Kleine, T. A long-lived magma ocean on a young Moon. *Sci. Adv.* **6**, eaba8949 (2020). 32695879 10.1126/sciadv.aba8949 PMC7351470

[18] Jacobson, S. A. et al. Highly siderophile elements in Earth’s mantle as a clock for the Moon-forming impact. *Nature* **508**, 84–87 (2014). 24695310 10.1038/nature13172

[19] Mezger, K., Maltese, A. & Vollstaedt, H. Accretion and differentiation of early planetary bodies as recorded in the composition of the silicate Earth. *Icarus* **365**, 114497 (2021).

[20] Thiemens, M. M., Sprung, P., Fonseca, R. O. C., Leitzke, F. P. & Münker, C. Early Moon formation inferred from hafnium–tungsten systematics. *Nat. Geosci.* **12**, 696–700 (2019). 39649009 10.1038/s41561-019-0398-3 PMC7617097

[21] Borg, L. E., Brennecka, G. A. & Kruijer, T. S. The origin of volatile elements in the Earth–Moon system. *Proc. Natl Acad. Sci. USA* **119**, e2115726119 (2022). 35165180 10.1073/pnas.2115726119 PMC8872726

[22] Kruijer, T. S., Archer, G. J. & Kleine, T. No ^182^W evidence for early Moon formation. *Nat. Geosci*. 10.1038/s41561-021-00820-2 (2021).

[23] Garrick-Bethell, I., Perera, V., Nimmo, F. & Zuber, M. T. The tidal-rotational shape of the Moon and evidence for polar wander. *Nature* **512**, 181–184 (2014). 25079322 10.1038/nature13639

[24] O’Reilly, T. C. & Davies, G. F. Magma transport of heat on Io: a mechanism allowing a thick lithosphere. *Geophys. Res. Lett.* **8**, 313–316 (1981).

[25] Spencer, D. C., Katz, R. F. & Hewitt, I. J. Tidal controls on the lithospheric thickness and topography of Io from magmatic segregation and volcanism modelling. *Icarus* **359**, 114352 (2021).

[26] Miyazaki, Y. & Stevenson, D. J. A subsurface magma ocean on Io: exploring the steady state of partially molten planetary bodies. *Planet. Sci. J.* **3**, 256 (2022).

[27] Cuk, M. & Stewart, S. T. Making the Moon from a fast-spinning Earth: a giant impact followed by resonant despinning. *Science* **338**, 1047–1052 (2012). 23076099 10.1126/science.1225542

[28] Tian, Z., Wisdom, J. & Elkins-Tanton, L. Coupled orbital-thermal evolution of the early Earth–Moon system with a fast-spinning Earth. *Icarus* **281**, 90–102 (2017).

[29] Rufu, R. & Canup, R. M. Tidal evolution of the evection resonance/quasi-resonance and the angular momentum of the Earth–Moon system. *J. Geophys. Res. Planets* **125**, e2019JE006312 (2020). 10.1029/2019je006266 PMC7545365 33042721

[30] Ćuk, M., Lock, S. J., Stewart, S. T. & Hamilton, D. P. Tidal evolution of the Earth–Moon system with a high initial obliquity. *Planet. Sci. J.* **2**, 147 (2021).

[31] Siegler, M. A., Bills, B. G. & Paige, D. A. Effects of orbital evolution on lunar ice stability. *J. Geophys. Res. Planets* **116**, E03010 (2011).

[32] Downey, B. G., Nimmo, F. & Matsuyama, I. The thermal–orbital evolution of the Earth–Moon system with a subsurface magma ocean and fossil figure. *Icarus* **389**, 115257 (2023).

[33] Tian, Z. & Wisdom, J. Vertical angular momentum constraint on lunar formation and orbital history. *Proc. Natl Acad. Sci. USA* **117**, 15460–15464 (2020). 32571906 10.1073/pnas.2003496117 PMC7355045

[34] Veeder, G. J., Matson, D. L., Johnson, T. V., Blaney, D. L. & Goguen, J. D. Io’s heat flow from infrared radiometry: 1983–1993. *J. Geophys. Res.* **99**, 17095–17162 (1994).

[35] Wilson, L. & Head, J. W. Generation, ascent and eruption of magma on the Moon: new insights into source depths, magma supply, intrusions and effusive/explosive eruptions (part 1: theory). *Icarus* **283**, 146–175 (2017).

[36] Brandon, A. D. et al. Re-evaluating Nd-142/Nd-144 in lunar mare basalts with implications for the early evolution and bulk Sm/Nd of the Moon. *Geochim. Cosmochim. Acta* **73**, 6421–6445 (2009).

[37] Shearer, C. K. et al. Thermal and magmatic evolution of the Moon. *Rev. Mineral. Geochem.* **60**, 365–518 (2006).

[38] Longhi, J. Experimental petrology and petrogenesis of mare volcanics. *Geochim. Cosmochim. Acta* **56**, 2235–2251 (1992).

[39] Borg, L. E., Gaffney, A. M. & Shearer, C. K. A review of lunar chronology revealing a preponderance of 4.34–4.37 Ga ages. *Meteorit. Planet. Sci.* **50**, 715–732 (2015).

[40] Whitaker, E. A. The lunar Procellarum Basin. In *Multi-ring Basins: Formation and Evolution; Proc. Lunar and Planetary Science Conference* 105–111 (Pergamon Press, 1981).

[41] Garrick-Bethell, I., Wisdom, J. & Zuber, M. T. Evidence for a past high-eccentricity lunar orbit. *Science* **313**, 652–655 (2006). 16888135 10.1126/science.1128237

[42] Miljković, K. et al. Large impact cratering during lunar magma ocean solidification. *Nat. Commun.* **12**, 5433 (2021). 34521860 10.1038/s41467-021-25818-7 PMC8440705

[43] Bottke, W. F., Walker, R. J., Day, J. M. D., Nesvorny, D. & Elkins-Tanton, L. Stochastic late accretion to Earth, the Moon, and Mars. *Science* **330**, 1527–1530 (2010). 21148387 10.1126/science.1196874

[44] Marchi, S., Canup, R. M. & Walker, R. J. Heterogeneous delivery of silicate and metal to the Earth by large planetesimals. *Nat. Geosci.* **11**, 77–81 (2018). 30984285 10.1038/s41561-017-0022-3 PMC6457465

[45] Zhu, M.-H. et al. Reconstructing the late accretion history of the Moon. *Nature* **571**, 226–229 (2019). 31292556 10.1038/s41586-019-1359-0

[46] Zahnle, K. J., Lupu, R., Dobrovolskis, A. & Sleep, N. H. The tethered Moon. *Earth Planet. Sci. Lett.* **427**, 74–82 (2015).

[47] Korenaga, J. Rapid solidification of Earth’s magma ocean limits early lunar recession. *Icarus* **400**, 115564 (2023).

[48] Ray, R. D., Eanes, R. J. & Chao, B. F. Detection of tidal dissipation in the solid Earth by satellite tracking and altimetry. *Nature* **381**, 595–597 (1996).

[49] Lainey, V., Arlot, J.-E., Karatekin, Ö. & van Hoolst, T. Strong tidal dissipation in Io and Jupiter from astrometric observations. *Nature* **459**, 957–959 (2009). 19536258 10.1038/nature08108

[50] Goldreich, P. & Soter, S. *Q* in the Solar System. *Icarus* **5**, 375–389 (1966).

[51] Farhat, M., Auclair-Desrotour, P., Boué, G. & Laskar, J. The resonant tidal evolution of the Earth–Moon distance. *Astron. Astrophys.* **665**, L1 (2022).

[52] Kleine, T. & Walker, R. J. Tungsten isotopes in planets. *Ann. Rev. Earth Planet. Sci.* **45**, 389–417 (2017). 30842690 10.1146/annurev-earth-063016-020037 PMC6398955

[53] Salmon, J. & Canup, R. M. Lunar accretion from a Roche-interior fluid disk. *Astrophys. J.* **760**, 83 (2012).

[54] Watts, A. B. *Isostasy and Flexure of the Lithosphere* (Cambridge Univ. Press, 2001).

[55] Wieczorek, M. A. et al. The crust of the Moon as seen by GRAIL. *Science* **339**, 671–675 (2013). 23223394 10.1126/science.1231530 PMC6693503

[56] Moore, W. B., Simon, J. I. & Webb, A. A. G. Heat-pipe planets. *Earth Planet. Sci. Lett.* **474**, 13–19 (2017).

[57] Carslaw, H. S. & Jaeger, J. C. *Conduction of Heat in Solids* (Oxford Univ. Press, 1986).

[58] Cherniak, D. J. & Watson, E. B. Pb diffusion in zircon. *Chem. Geol.* **172**, 5–24 (2001).

[59] Meyer, J., Elkins-Tanton, L. & Wisdom, J. Coupled thermal–orbital evolution of the early Moon. *Icarus* **208**, 1–10 (2010).

[60] Murray, C. D. & Dermott, S. F. *Solar System Dynamics* (Cambridge Univ. Press, 2000); 10.1017/CBO9781139174817.

[61] Citron, R. I., Smith, D. E., Stewart, S. T., Hood, L. L. & Zuber, M. T. The South Pole–Aitken Basin: constraints on impact excavation, melt, and ejecta. *Geophys. Res. Lett.* **51**, e2024GL110034 (2024).

[62] Jones, M. J. et al. A South Pole–Aitken impact origin of the lunar compositional asymmetry. *Sci. Adv.* **8**, eabm8475 (2022). 35394845 10.1126/sciadv.abm8475 PMC8993107

[63] Snape, J. F. et al. Ancient volcanism on the Moon: insights from Pb isotopes in the MIL 13317 and Kalahari 009 lunar meteorites. *Earth Planet. Sci. Lett.* **502**, 84–95 (2018).

[64] Melosh, H. J. *Impact Cratering: A Geologic Process* (Oxford Univ. Press, 1989).

[65] Croft, S. K. Cratering flow fields: implications for the excavation and transient expansion stages of crater formation. *Lunar Planet. Sci. Conf. Proc.* **3**, 2347–2378 (1980).

[66] Barnhart, C. J. & Nimmo, F. Role of impact excavation in distributing clays over Noachian surfaces. *J. Geophys. Res. Planets* **116**, E01009 (2011).

[67] Zahnle, K., Schenk, P., Levison, H. & Dones, L. Cratering rates in the outer Solar System. *Icarus* **163**, 263–289 (2003).

[68] Robbins, S. J. A new global database of lunar impact craters >1–2 km: 1. Crater locations and sizes, comparisons with published databases, and global analysis. *J. Geophys. Res. Planets* **124**, 871–892 (2019).

[69] Potter, R. W. K., Collins, G. S., Kiefer, W. S., McGovern, P. J. & Kring, D. A. Constraining the size of the South Pole–Aitken Basin impact. *Icarus* **220**, 730–743 (2012).

[70] Ganguly, J. & Tirone, M. Relationship between cooling rate and cooling age of a mineral: theory and applications to meteorites. *Meteorit. Planet. Sci.* **36**, 167–175 (2001).

